# Determinants of Public Attitudes to Genetically Modified Salmon

**DOI:** 10.1371/journal.pone.0086174

**Published:** 2014-01-29

**Authors:** Latifah Amin, Md. Abul Kalam Azad, Mohd Hanafy Gausmian, Faizah Zulkifli

**Affiliations:** 1 Centre for General Studies, Universiti Kebangsaan Malaysia, Bangi, Selangor, Malaysia; 2 Department of Agricultural Extension, Khamarbari, Farmgate, Dhaka, Bangladesh; 3 Faculty of Science and Technology Universiti Kebangsaan Malaysia, Bangi, Selangor, Malaysia; TGen, United States of America

## Abstract

The objective of this paper is to assess the attitude of Malaysian stakeholders to genetically modified (GM) salmon and to identify the factors that influence their acceptance of GM salmon using a structural equation model. A survey was carried out on 434 representatives from various stakeholder groups in the Klang Valley region of Malaysia. Public attitude towards GM salmon was measured using self-developed questionnaires with seven-point Likert scales. The findings of this study have confirmed that public attitudes towards GM salmon is a complex issue and should be seen as a multi-faceted process. The most important direct predictors for the encouragement of GM salmon are the specific application-linked perceptions about religious acceptability of GM salmon followed by perceived risks and benefits, familiarity, and general promise of modern biotechnology. Encouragement of GM salmon also involves the interplay among other factors such as general concerns of biotechnology, threatening the natural order of things, the need for labeling, the need for patenting, confidence in regulation, and societal values. The research findings can serve as a database that will be useful for understanding the social construct of public attitude towards GM foods in a developing country.

## Introduction

Biotechnology has become an important field in the global market. Genetically modified (GM) food involves the deliberate modification of plants and animals' genetic material using innovative recombinant DNA technology [1]. Genetic engineering techniques have been envisaged as an opportunity to improve food production to fulfill consumer preferences for improved quality and diversity. The modification of food genetically has the potential to increase yields and could lower the price of food, which would boost productivity in farming and increase the supply of food for the world's rapidly growing population [2]. Although genetic modification technology holds the promise to increase food security in developing countries, negative public acceptance can affect its adoption [3]. The public's main concerns relate to the uncertainties and possible negative effects of genetically modified organisms (GMOs) on human health and the environment [4], [5]. Despite the associated benefits of genetic engineering of food, its successful adoption can only become a reality if consumers accept the end-products. The future advancement of gene technology very much depends on public acceptance.

Aquaculture has been reported as the fastest growing food industry worldwide, and salmon farming has been identified as the fastest growing sector in aquaculture [6]. GM salmon may become an important source of protein to meet the growing demand of the growing global population. According to Cowx et al. [7], around 50 species/traits of animals have been modified genetically. The modifications mostly involved fish species such as Atlantic salmon, common carp and tilapia. The popular traits engineered include growth rate improvements, resistance to diseases, efficiency of feed conversion, low oxygen level tolerance, resistance to cold and freezing, and the ability to utilize low cost and non-animal protein based diets [8], [9], [10]. These traits could be developed by genetic modification of fish, which would in turn make a great contribution to the modern fish sector. GM salmon was the first genetically modified animal approved for human consumption in the United States [11]. The GM Atlantic salmon were engineered with either antifreeze protein genes or salmon growth hormone gene constructs. The GM Atlantic salmon inserted with the type III antifreeze protein were able to survive in water at temperatures of below 0°C [12]. The growth rate of GM Atlantic salmon, which was inserted with the growth hormone gene from Chinook salmon, is higher than conventional fish and can reach their commercial size in one third of the time required for non-transgenic salmon [13]. GM salmon has great potential to provide a sustainable food alternative, and could help solve economic and environmental constraints in aquaculture farming [14].

Public understanding, perception, and acceptance of genetic modification (GM) technologies and their products would either promote or hamper their adoption and commercialization [15]. Wynne [16] stressed the existing of unacknowledged problem of our contemporary scientific culture that are not being recognized and address. The experts have always argued that public concerns are based on ignorance and misunderstanding of science. In reality, most people are less aware of the scientific nature of a risk, and are much more concerned with broader, qualitative attributes [17]. Acceptance involves an individual's attitude to certain political issues such as those involving technological innovation [18]. People's attitude towards a new technology, such as GM technology, is determined by several interrelated factors including perceived risks and perceived benefits, social values, and trust in key actors or institutions representing and governing these technologies [19], [20], [21] as well as general attitudes and personal values [22].

Public attitudes towards GM technology have been found to differ across various cultures and geographical regions worldwide [19], [23] as well as across different types of applications [20], [24], [25]. Bauer [26] stressed that GM foods were found to be more controversial and were put into the negative light compared to medical biotechnology. Consumers in developed countries worldwide were not very supportive of the development of GM salmon for food. According to Chern et al. [27], consumers in the United States and Norway were willing to pay more for non-GM salmon than for GM salmon but consumers in the United States were more favorable towards GM salmon compared with their counterparts in Norway. Haro [28] reported that the majority of life-science students at university in Norway were not keen on buying GM transgenic salmon even though it has benefits such as increased nutrition and disease resistance. In Scotland, the salmon industry has been reported to have voted against GM salmon [6]. A more recent survey in 2010 by the Lake Research Partners [29] in the United States revealed that 78% of respondents did not support the approval of GM salmon for human consumption by the United States Federal Food and Drug Administration. Menozzi et al. [14] revealed through qualitative scenario analysis that the majority of experts in Europe, Canada and Chile did not believe that the GM of salmon will be an important technical innovation.

Several studies have tried to identify the factors determining consumers' acceptance of GM salmon in Europe and the United States. The majority of the studies on willingness to pay for GM salmon concluded that price was the major factor affecting consumer choice [27], [30], [31], [32]. Additional determinants identified were tangible benefits [27], [30], [31], health risks [27], [30], nature of transfer and environmental concerns [31], and knowledge and education [32]. Nep and O'Doherty [33] analyzed public deliberation on GM salmon in Canada and found that labeling was inadequately addressed in Canada's regulatory framework. Although some of the factors determining consumers support of GM salmon have been identified in the earlier studies [14], [27], [28], [30], [31], [32], [34], the various factors were not derived from a similar study or similar respondents. Moreover, focus-group studies [14], [31], [32], [34] have the limitation of small samples. There are other factors that have been cited as important in influencing consumers' acceptance of GM foods that have not been included in the above studies related to GM salmon. The factor of religious acceptance has been shown to be important for Malaysians [35], [36]. Other general attitudinal factors such as confidence in regulation, attitude to patenting, moral aspects (threatening natural order), societal value (nature versus materialism), attitude to labeling and general concerns and the promise of modern biotechnology are also included in this study to assess their influence on the acceptance of GM salmon. Their importance will be highlighted in the theoretical framework section of this paper.

There has been no attempt to use structural equation modeling (SEM) to assess factors influencing the acceptance of GM salmon in past studies. The use of SEM has several advantages over other techniques. It is an advanced multivariate technique that assesses multiple dependence relationships between variables simultaneously [37], [38], allows the modelling and prediction of relationships between construct variables in a hypothesized manner and has the ability to suggest novel hypotheses originally not considered. It helps to specify hypotheses and operationalize constructs more precisely and ensures the reliability of measures in the testing of hypotheses in ways beyond the averaging of multi-measures of constructs [39], [40], [41]. SEM has the ability to model latent variables, specify errors, correct measurement error, analyze covariance structures and construct complete theories simultaneously [40].

There have been only limited studies on Asian attitudes towards GM foods and GM salmon and none on the identification of factors determining the public acceptance of GM salmon. Our earlier publications identified the dimensions of public attitude towards modern biotechnology in general [42] and several applications of gene technology in plants [43], [44]. This paper further analyzes the Malaysian attitude towards GM animals as past studies have shown that people were less supportive of genetic modification of animals compared with that of plants [25]. The relationships between the various factors (dimensions) and their influence on the acceptance of GM application, which has not been determined in earlier studies [43], [44], will also be assessed in this paper. The objective of this paper is to assess the Malaysian stakeholders' attitude towards GM salmon and to identify the factors that influence their acceptance of GM salmon using SEM.

### Theoretical Framework

The theoretical framework of this study has been developed based on the attitude model towards biotechnology applications proposed by Pardo et al. [45] and Bredahl's Attitude Model towards GM foods [19], which was based on Fishbein's Multi-attribute Attitude Model [46]. The model begins with potential causes that are known to affect attitudes. The variables are arranged according to their assumed influence on the subsequent variables. The model shows that specific attitude towards modern biotechnology applications is influenced by the more general attitudinal and values variables [19], [22], [47] and the relationships between the variables were hypothesized based on earlier studies. The magnitude of any influence or association between two variables will be determined by the regression weights from structural equation modeling (SEM) analysis.

#### Specific attitude

In the model, the overall attitude (encouragement) towards a specific application or product of modern biotechnology such as GM salmon is determined by the specific perceptions of risks and benefits [48], [49], familiarity, and religious acceptance of GM salmon. According to Campbell et al. [50], evaluation of any complex object is affected by extraneous concerns, which are also known as general attitude or schemas [46]. Since GM foods are considered new to the food arena, people may have difficulties in configuring them in their minds, so it is expected that attitudes towards them would be influenced by more general attitudes and values that have been embedded in their minds [19], [22], [47], [49]. So the specific attitudinal variables: familiarity, perceived risks and benefits, and religious acceptability of GM salmon are given causal interpretations by several general attitudinal and values variables such as general promise and concerns of modern biotechnology [45], societal value [24], moral concerns [51], confidence on regulation [49], [52] and the need for labelling [20] and patenting of GM products [53].

Perceived benefit and perceived risk have been identified by many researchers as important variables for the measurement of public attitudes towards GM technology [19], [54], [55], [56], [57]. Perception of benefits and risks are complex and difficult to conceptualize separately. Several studies have suggested that perceived risk and benefit are not independent [54], [56], [58], but that people instead tend to judge an inverse relationship between perceived risks and perceived benefit. Fischhoff et al. [59] also reported a consistent association between perceived benefit and acceptable level of risk. Perception of benefits revolves around producers, consumers, health, and societal issues, while risks are extended to include long-term effects on human health, the environment, and societal and moral issues [56], [57]. The encouragement variable in this study reflects support towards the GM product or the overall attitude or acceptability of a GM application. This dimension has been introduced and repeatedly used by the Eurobarometer time series surveys [60], [61], [62] and other researchers [55], [63].

According to Verdurme and Viaene [64] and Costa-Font et al. [20], a consumer's knowledge about GM technology can play a role in determining their risk and benefit perception towards GM foods. On the other hand, other studies have proved that acceptance of modern biotechnology by the public may not be related to awareness at all. Frewer et al. [65], [66] stressed that people were able to judge whether GM technology is useful or risky regardless of whether they were aware of the technology. Chen and Li [49] further showed that factual knowledge about gene technology was not related to the perceived benefits of GM foods and was only weakly related to their perceived risks. Bauer [67] concluded that in controversial issues, knowledge is not related at all to positive attitudes but attitude that are based on knowledge will be held more strongly. Bucchi and Neresini [68] also reported that the better informed public will be more likely to be more critical in evaluating modern biotechnology applications. Costa-Font et al [20] suggested the differentiation between ‘objective knowledge’, which refers to people's real knowledge about GM food, and ‘subjective knowledge’ which ascribe people's perception of what they know about GM foods. Subjective knowledge has been reported to be more related to general attitudes, values and acceptance of GM foods compared to objective knowledge [20], [69]. The variable, familiarity has been recognized and demonstrated to be an important dimension in risk perception studies [56], [58], [70], [71]. Because familiarity with GM food was found to have a positive effect on its acceptance [72] as well as it represent subjective knowledge, the dimension of familiarity is included in this model. Kirk et al. [71] suggested that familiarity comprised several items related to the ease of identifying a product that contains risky substances, whether the risk is known to science and whether people have control over eating a particular product.

Furedi [73] stressed that risk perception at the individual and societal level are affected by moral values. If a product was perceived as worthy and not having moral concerns, people would be willing to accept its associated risks to a certain extent. Gaskell et al. [62] proposed moral acceptability as the veto predictor for the encouragement of several biotechnology applications. People from the United States were also found to use moral reasoning in their perception of six biotechnology applications [63]. A study by Einseidel [55] showed that moral acceptance was the strongest predictor for encouragement of animal cloning. Chen and Li [49] have suggested that an additional attitudinal indicator related to acceptance of GM food from religious beliefs or customs should be included in future studies. Amin et al. [35] reported that the stakeholders in Malaysia have claimed to have high attachment to their religion. The Canadian Trade Commissioner Service [36] also highlighted that Malaysians of all faiths are usually deeply attached to their religion. So in this study, the variable of religious acceptance has been added to represent the specific moral acceptance of GM food in Malaysia. The more general moral concerns have been conceptualized as threatening the natural order of things, because past studies have associated the moral aspects of GM products with interfering with nature [51] and being seen as playing God [74].

#### General attitude and values

GM foods are new to the food scene and people are expected to have difficulties in conceiving them as concrete product entities. Instead people tended to apply their more general attitudes to evaluate new and unfamiliar foods [49]. The general attitudinal variables included in the theoretical model of this study were general promise and concern of modern biotechnology, biotechnology regarded as threatening natural order of things, attitude towards labeling (the need for labeling), confidence in regulation, attitude to patenting, and societal value.

Pardo et al. [45] have identified general promise and reservations (concerns) as two important schemas for the European people's attitude towards biotechnology. They reported that the general promise schema as the strongest predictor of perceived benefits of biotechnology application and inversely related to perceived risk. On the other hand, the reservation schema was found to be positively related to perceived risk of biotechnology application. Past studies have shown that moral concerns as an important determinant for the support of GM foods [62]. In this study, the specific moral concerns have been conceptualized as religious acceptability. Some researchers have highlighted that modern biotechnology in general has been seen as threatening the natural order of living things [75], [76]. Taking the lead from the structure proposed by Pardo and colleagues [45], a general schema for moral concerns is included in this framework and termed as biotechnology regarded as threatening the natural order of things.

Societal value (nature versus materials) has been shown to predict encouragement of six biotechnology applications [24] and has exerted considerable influence on both perceived risk magnitude and risk acceptance of technological risks [77]. People who are more inclined towards materials were found to be more supportive of biotechnology applications compared to those with nature inclined attitude.

Most of the time people cannot directly assess the benefit and risks of GM food and they have to depend on information provided by experts or others resources. Therefore consumers have to depend on experts and institutions in managing risks associated with technologies [53]. Past studies confirmed that consumers' trust in science and institutions involved in gene technology regulation is related to their perception of risks and benefits of gene technologies [49], [78], [79]. Bucchi and Neresini [52] highlighted that inadequate governance of innovation led to public rejection of some biotechnology applications. Past studies have proved the association of trust on related institutions with perceived benefits of GM foods but there has been no study to test the effect of confidence on GMOs regulation. In this framework, confidence on regulation is incorporated to assess its influence on the specific attitude variables.

Potrykus [80] reported that the patenting right of scientists has become a serious issue for GM technology. Uzogara [81] has highlighted the possibilities that patenting might allows big corporations to monopolize GM plants and animals besides patenting is said to violate the sanctity of life. Many critics also oppose the fact that seeds are now regarded as propriety products, moreover with the ‘terminator gene’ technology which renders the seeds sterile [82]. The farmers are force to buy new seeds each year from multinational companies instead of sowing seeds from previous years' harvest. Labeling is an important source of information so that consumers know the ingredients in the food they consume [20], [83], [84]. Although the main function of labels is to provide information but labelling may also function as a cue for product safety [83], [85]. Although some consumers may use labelling to avoid biotechnology products, others may perceive the explicit labelling as a sign of the manufacturers' confidence in the product's safety. Wansink & Kim [83] highlighted the important of providing consumers a sense of control over their choices. When their confusion about what and how to choose diminishes, the consumers will be more comfortable and confident in accepting biotechnology. There has been no attempt to study the influence of these two variables in earlier models on attitude towards GM foods. Due to their importance, attitude towards patenting and labeling were included in the model to assess their influence on the specific attitude towards GM salmon.

## Materials and Methods

The research data were obtained through face-to-face surveys of 434 adults (aged 18 years and above) residing in the Klang Valley region from August 2009 to February 2010. The Klang Valley was chosen to be the study area because it is the center of Malaysia's economic and social development, and because the people who reside there meet the study's requirement of diversity among respondents. The respondents were selected based on stratification according to stakeholders' groups. The groups selected included producers (5.8%), policy makers (9.0%), scientists (7.4%), non-governmental organizations (6.0%), media (6.7%), university students (10.1%), religious scholars (Muslim 9.9%, Christian 7.8%, Buddhist 7.4%, Hindu 7.8%) and consumers (22.1%). Since the respective populations for the stakeholders involved were unknown, the respondents were chosen using stratified purposive sampling as recommended by Monroe & Monroe [86]. This technique allows comparisons among respondents from different stakeholder groups that might otherwise be underrepresented if random sampling was used. Sixty-two per cent of the respondents were female and 38% male, ages ranged from 18–64 years, the majority (62.9%) of respondents had tertiary level education, and 23.5% had pre-university education or diploma holders while the remaining 13.6% had at least secondary level education. The survey approach used was based on the conventional multiple indicator proposed by Kelly [87] to make it easier for the respondents to answer and to reduce measurement errors. The questionnaires were conducted face-to-face by trained graduate enumerators. Before answering, the respondents were first briefed with an introduction to basic concepts of genetic engineering and development of GM salmon. Participation of the respondents in this study was voluntary and anonymous. Ethic approval was not needed for this study as under the Guidelines for Ethical Review of Clinical Research or Research involving human subjects, Ministry of Health Malaysia [88], research involving questionnaires with no collection of identifiable private information would be exempted from the Medical Review and Ethics Committee, Ministry of Health Malaysia. Although informed consent was also exempted for research in this category, verbal informed consent was asked before the respondents answered the questionnaires and was recorded by the enumerators. The research data could not be deposited in a public resource as this project is funded by the Malaysian government but the data is available upon request from the corresponding author.

### Instrument

The multi-dimensional instrument measuring perceptions towards the ethical aspects of modern biotechnology used in this study was developed based on past studies. The instrument incorporated seven general ethical aspects: threatening the natural order of living things [75], [89], [90], [91], general concerns [92], patenting needs [80], [92], general promise [50], [93], [94], the need for labeling [95], [96], [97], confidence in biotechnology regulation [98], and societal value [76]. It also included five specific ethical aspects: familiarity [71], [99], [100], perceived risks [76], [93], [97], [101], perceived benefits [102], [103], religious acceptance [87], [104], and encouragement [24]. All items were measured on seven-point Likert scales.

Threatening the natural order of living things (α = 0.814) comprised the average mean response to three items: modern biotechnology application is considered blasphemous, the work of scientists modifying the genetic characteristics of living organisms is considered over the limit, and modern biotechnology interferes with the natural integrity of living organisms. Each item was measured on a seven-point scale, ranging from 1 (strongly disagree with the statement) to 7 (strongly agree with the statement). A higher score indicated a greater perceived threat to the natural order of living things.

Patenting need (α = 0.852) was measured by three items: the biotechnology industry needs to be encouraged to patent its biotechnology innovations, intellectual property rights are the best reward to cover modern biotechnology's developmental costs by industry, and patenting is needed to protect the scientist's intellectual property rights. Each item was measured on a seven-point scale, ranging from 1 (strongly disagree with the statement) to 7 (strongly agree with the statement). A higher score indicated greater patenting needs. Societal value (α = 0.813) was assessed based on the respondents' preferences for three bipolar statements concerning nature and materials value [76]. Each item was measured on a seven-point scale, ranging from 1 (strongly preferred nature value) to 7 (strongly preferred material value).

Confidence in biotechnology regulation (α = 0.703) comprised two items: the regulatory action on experimental failure of GMOs is adequate in protecting the safety of Malaysian society, and the government department involved in modern biotechnology regulation has monitored the safety of modern biotechnology products efficiently. Each item was measured on a seven-point scale, ranging from 1 (strongly disagree with the statement) to 7 (strongly agree with the statement). A higher score indicated a greater level of confidence in biotechnology regulation.

The need for labeling (α = 0.861) was assessed by four items: labeling of biotechnology products is important for those who have allergies to certain foods, labeling is essential to differentiate between GM products and non-GM products, labeling is important to provide information regarding modern biotechnology products, and labeling of modern biotechnology products is the responsibility of the producer. Each item was measured on a seven-point scale, ranging from 1 (strongly disagree with the statement) to 7 (strongly agree with the statement). A higher score indicated a greater perceived need for labeling.

General concerns (α = 0.798) comprised three items: modern biotechnology may increase human fatality, consuming modern biotechnology products may inhibit normal growth among children, and modern biotechnology products may cause the transfer of animal disease to humans. Each item was measured on a seven-point scale, ranging from 1 (strongly disagree with the statement) to 7 (strongly agree with the statement). A higher score indicated a greater perceived risk to human health.

General promise (α = 0.724) was measured using two items: modern biotechnology can improve Malaysia's economy, and modern biotechnology can reduce starvation in poor countries. Each item was measured on a seven-point scale, ranging from 1 (strongly disagree with the statement) to 7 (strongly agree with the statement). A higher score indicated greater perceived benefits.

Familiarity (α = 0.685) comprised two questions: ‘How easy is it for you to know whether is it good or bad to consume the following modern biotechnology products?’ and ‘Are the effects of consuming the following modern biotechnology products well known?’ Each item was measured on a seven-point scale, ranging from 1 (not easy at all for the first two items/strongly disagree for the last item) to 7 (very easy for the first two items/strongly agree for the last item). A higher score indicated a greater familiarity with biotechnology products.

Perceived benefits if it is not developed (α = 0.816) was assessed by three items: the potential of the application to boost the country's economy, the potential to improve the quality of life in Malaysian society, and the potential to improve the lives of farmers and breeders. Each item was measured on a seven-point scale, ranging from 1 (strongly disagree) to 7 (strongly agree). A higher score indicated greater perceived benefits if biotechnology is not developed.

Perceived risks (α = 0.822) was measured by four items: the following application might reduce the status of living things to machines; ‘How worried are you about potential risks of the following food to your health?’; any harmful effects from consuming the following food will only manifest itself after a long term duration; and any danger from the following food could cause a major catastrophe to the Malaysian society. Each item was measured on a seven-point scale, ranging from 1 (strongly disagree for the first item/not worried at all for the second item) to 7 (strongly agree for the first item/very worried for the second item). A higher score indicated greater perceived risks.

Religious acceptance (α = 0.868) was measured by two items: the application can be accepted by my religion, and the application can be accepted by my customs. Each item was measured on a seven-point scale, ranging from 1 (not accepted at all) to 7 (strongly accepted). A higher score indicated greater religious acceptance.

Encouragement (α = 0.861) comprised three items: more intensive research should be encouraged to develop the application; the government should provide more financial support to researchers and industries in developing the application; and ‘How far should the following applications be encouraged?’ Each item was measured on a seven-point scale, ranging from 1 (strongly disagree) to 7 (strongly agree). A higher score indicated greater ethical acceptance.

### Statistical Analysis

Structural equation modeling (SEM) is known to be powerful multivariate analysis techniques to detect causal relationships among variables [105], [106], [107]. The structural equation model in this study was developed on the basis of previous research findings as well as correlations among variables. The model was designed according to the assumed influenced variables as proposed by Pardo et al. [45]. A single step SEM analysis as recommended by Hair et al. [108] was carried out to estimate the measurement and structural model using AMOS version 5.0 software with maximum likelihood function.

#### Testing of the measurement model

Before testing the proposed model with SEM, Gursory and Rutherford [109] recommended the assessment of the unidimensionality of each construct to ensure that each set of indicators has only one underlying trait or construct in common. In this study, unidimensionality of the overall measuring instrument was assessed using confirmatory factor analysis (CFA) and Cronbach's alpha. Only items or indicators with factor loadings minimally 0.30 [110] or item-total correlation at least 0.3 [111] were considered acceptable.

Table 1 shows the results of CFA for the measurement scale of attitude towards GM salmon using maximum likehood estimation. The analysis yielded 12 meaningful item groupings or constructs with strong unambiguous loadings. The three types of reliabilities measured in this paper are internal consistency (Cronbach alpha), item reliability and construct reliability. The Cronbach's alpha coefficients for the majority of the constructs were considered good (above 0.70; Table 1). The corrected item-total correlations for all items in each dimension were very good (correlation coefficients greater than 0.5; Table 1). The construct reliability is represented by the composite reliabilities and the variance extracted. From Table 1, it can be seen that the composite reliabilities for all constructs were above 0.7 and the variance extracted were all above 0.50, indicating good construct reliability [108]. The convergent validity was assessed by the factor loadings and composite reliability [108]. The standardized loadings of all factors were greater than 0.7, and the composite reliabilities for all factors were also above 0.7, indicating good convergent validity (Table 1).

**Table 1:** Measurement scales, reliability, and validity.

Factors and Items	Corrected item total correlation	α	Standardized factor loading	Composite reliability	Average Variance Extracted (AVE)
**Threaten natural order**					
1. Application considered blasphemous	0.626	0.814	0.702	0.794	0.597
2. The work of scientist is considered over limit	0.674		0.757		
3. Interferes with natural integrity	0.697		0.852		
**Patenting**					
4. Industry encouraged to patent	0.704	0.852	0.792	0.853	0.660
5. Reward to cover development cost	0.725		0.817		
6. Protects the scientist rights	0.739		0.829		
**Societal value**					
7. Use nature for prosperity versus preserving nature	0.714	0.809	0.860	0.811	0.591
8. Accept risk to attain prosperity versus not striving for progress	0.622		0.702		
9. Place economic growth above environmental protection versus the opposite	0.637		0.736		
**Confidence on regulation**					
10. Regulatory action GMO failure adequate	0.543	0.703	0.663	0.713	0.556
11. Efficient monitoring by government	0.543		0.821		
**The need for labeling**					
12. Important for those who have allergy	0.679	0.861	0.733	0.873	0.638
13. To differentiate between GM and non-GM	0.807		0.915		
14. To provide information	0.806		0.912		
15. Labeling is the responsibility of the producer	0.559		0.591		
**General concern**					
16. Increase human fatality	0.645	0.798	0.749	0.800	0.574
17. Inhibit normal growth among children	0.697		0.843		
18. Cause transfer of animal disease to human	0.590		0.671		
**General promise**					
19. Improve Malaysia's economy	0.570	0.724	0.822	0.727	0.573
20. Reduce starvation in poor countries	0.570		0.686		
**Religious acceptance**					
21.Accepted by religion	0.751	0.858	0.899	0.858	0.752
22.Accepted by custom	0.751		0.835		
**Familiarity**					
23. Easy to know	0.521	0.685	0.696	0.685	0.522
24. Easy to judge	0.521		0.748		
**Perceived Benefit**					
25.Enhance country's economy	0.701	0.816	0.831	0.820	0.605
26.Enhance society's quality of life	0.693		0.814		
27.Improve farmer's & breeders life	0.613		0.68		
**Perceived Risk**					
28. Equal status between non-living and living organism	0.513	0.822	0.568	0.827	0.550
29. Worry about potential risk to health	0.674		0.779		
30.Harmful effects manifest after a long term	0.702		0.787		
31. Could cause major catastrophe	0.706		0.808		
**Encouragement**					
32. More intensive research and development	0.523	0.794	0.590	0.803	0.583
33. The government should provide more financial support	0.712		0.835		
34. Overall encouragement	0.684		0.839		

#### Testing of the structural model

**Figure 1:**
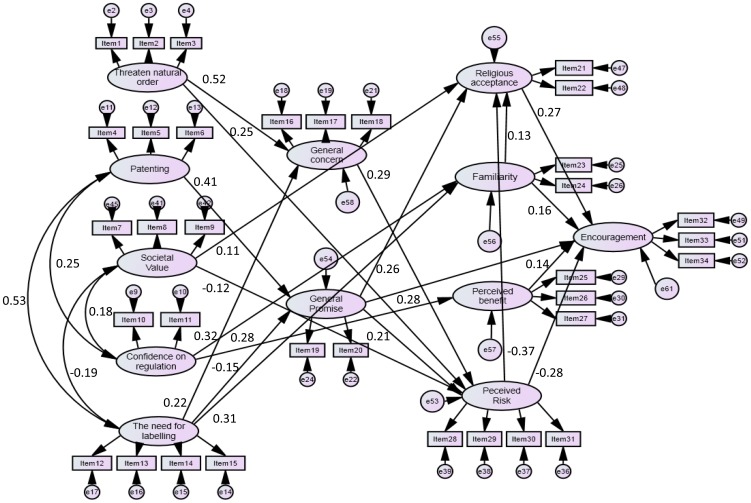
Structural equation model of public attitude to GM salmon. Significant pathways are represented by solid lines among the factors; relationships among the factors are given as standardized estimates; items 1–3 are indicators for the threatening of natural order; items 4–6 are indicators for attitude towards patenting; items 7–9 are indicators for societal value; items 10 and 11 are indicators for confidence in regulations; items 12–15 are indicators for the need for labeling; items 16–18 are indicators for general concerns about biotechnology; items 19 and 20 are indicators for the general promise of biotechnology; items 21 and 22 are indicators for religious acceptance of GM salmon; items 23 and 24 are indicators for familiarity with GM salmon; items 25–27 are indicators for the perceived benefits of GM salmon; items 28–31 are indictors for the perceived risk of GM salmon; items 32–34 are indicators for the encouragement of GM salmon; e1–e52 are measurement errors; e53–e61 are errors in equations. Indicators are described in Table 1.

After confirming the unidimensionality of the measurement scale, SEM was carried out for the proposed model. A single step SEM analysis as proposed by Hair et al. [112] was carried out to estimate simultaneously the structural and measurement models using AMOS version 5.0 software with maximum likehood function. The model generation strategy as recommended by Joreskog and Sorbom [113] was used to specify the model in this study but the modifications of the nested models were only carried out when they were substantively meaningful. A series of five nested models were tested to identify the best model for attitude towards GM salmon (Table 2). The first model was developed and specified according to the theoretical framework described earlier. Forty-two hypotheses were formulated from significant bivariate correlations at p<0.05 among the 12 factors as can be seen in Table 3. In the second model, nineteen of the forty-two proposed paths (parameters) that were non-significant were eliminated. In SEM, it has been suggested to remove non-significant parameters of the original model and to add additional paths suggested by the modification index to improve the model fit as long it does not contradict with theory [109], [114]. Additionally, Hair et al. [108], [110] recommended the removal of items with large standardized residual covariances to increase model fit. Eight items with large standardized residual covariances were deleted in the third model. In the fourth model, an additional path suggested by the modification index was included and three additional items with large residuals were further removed in the fifth model. Only 24 hypothesized paths that were statistically significant at the 0.05 probability level or lower were retained in the final model (Figure 1). According to Hair et al. [110] and Arbuckle and Wothke [115], a well fitting model should have goodness-of-fit index (GFI), comparative fit index (CFI) and Tucker–Lewis index (TLI) values greater than 0.90, and a root-mean-square error of approximation (RMSEA) value of less than 0.05 supported with a narrow confidence interval. The final model was found to have a good fit with χ^2^/df = 1.565, CFI of 0.954, GFI of 0.902, TLI of 0.948 and RMSEA of 0.037 with a 90% confidence level in the range of 0.032–0.041.

**Table 2:** Model comparison.

Fit Index	Model 1	Model 2	Model 3	Model 4	Model 5
χ^2^_df_	1688.98_903_	1722.61_923_	1003.64_606_	998.31_605_	787.02_503_
χ^2^/df	1.871	1.867	1.656	1.650	1.565
CFI	0.91	0.908	0.942	0.942	0.954
GFI	0.85	0.847	0.887	0.887	0.902
TLI	0.901	0.902	0.936	0.937	0.948
RMSEA	0.045	0.045	0.039	0.039	0.037
(confidence interval)	(0.042–0.049)	(0.042–0.049)	(0.035–0.044)	(0.035–0.043)	(0.032–0.041)

**Table 3:** The correlation matrix among the factors in the research model.

	Threaten natural order	Confidence on regulation	Patenting	Labelling	Societal value	General concern	General promise	Familiarity	Perceived benefit	Perceived risk	Religious acceptance	Encouragement
Threaten natural order	1											
Confidence on regulation	−0.059	1										
Patenting	−0.087	0.149**	1									
Labelling	0.064	−0.043	0.491**	1								
Societal value	−0.037	0.202**	−0.041	−0.199**	1							
General concern	0.458**	−0.062	0.082	0.195**	−0.091	1						
General promise	−0.062	0.035	.449**	0.440**	−0.099*	−0.014	1					
Familiarity	0.054	0.275**	−0.066	−0.109*	0.143**	−0.05	−0.03	1				
Perceived benefit	0.095	0.194**	0.101*	0.029	0.126**	−0.051	0.111*	0.139**	1			
Perceived risk	0.395**	−0.097*	−.138**	0.029	−0.134**	0.344**	−0.175**	−0.069	0.069	1		
Religious acceptance	−0.204**	0.097*	.232**	0.119*	0.133**	−0.103*	0.252**	0.106*	0.087	−0.394**	1	
Encouragement	−0.155**	0.175**	.291**	0.194**	0.083	−0.120**	0.343**	0.203**	0.192**	−0.394**	0.462**	1

* p<0.05 ;

** p<0.01.

## Results and Discussion

### Attitude

Overall, the Malaysian stakeholders in the Klang Valley were more inclined towards nature (mean score of 3.35). They perceived modern biotechnology as moderately threatening to the natural order of things (mean score of 3.75), recognized the high promise that modern biotechnology could provide to society (mean score of 5.38) and highly acknowledged the patenting rights of the scientists and producers (mean score of 5.28) (Table 4). However, they also professed their moderate concerns about modern biotechnology (mean score of 4.49) and expressed a strong need for the proper labeling of modern biotechnology products (mean score of 5.74). They exhibited a moderate level of confidence in government regulations on modern biotechnology (mean score of 4.12).

**Table 4:** General attitudes.

Dimension	Mean score ± Standard deviation	Interpretation
Threatening of natural order	3.75±1.23	Moderate
Attitude towards patenting	5.28±1.17	High
Societal value (nature versus materialism)	3.35±1.32	Moderate
Confidence in regulation	4.12±1.08	Moderate
Need for labeling	5.74±1.12	High
General concern about modern biotechnology	4.49±1.18	Moderate
General promise of modern biotechnology	5.38±1.08	high

low: 1–2.99, moderate: 3.00–5.00, high: 5.01–7.00.

The respondents claimed that they were not particularly familiar with GM salmon (mean score below the mid-point value of 4.0) (Table 5). This finding is not surprising as modern biotechnology has been labeled as being ‘novel’ and ‘complex’ with only a moderate level of awareness and knowledge among the public [116], no mandatory labeling of modern biotechnology products in Malaysia and limited periodic coverage on modern biotechnology issues in the Malaysian general mass media [44]. This situation is not unique to Malaysians. The public in the United Kingdom was also found to have low familiarity with GM foods [71]. The Taiwanese were reported to eat many GM foods but they did not know it [30] and the population in the United States also did not realize that modern biotechnology has been part of their foods [117]. Demirci [118] found that only 49% of their respondents thought they had seen GM foods at supermarkets while shopping and only 41% expressed that they had consumed GM foods.

**Table 5:** Comparison of attitudes to GM salmon and GM crops.

Dimension	GM salmon (fish to fish gene transfer to enhance growth)	Golden rice (plant to plant gene transfer to increase vitamin A) [119]	GM rice (animal to plant gene transfer to increase vitamin C) [43]
Familiarity	3.20±1.15^2^	3.41±1.25^1^	3.05±1.20^3^
Perceived risk	4.07±1.20^2^	3.85±1.31^3^	4.79±1.24^1^
Perceived benefit	3.90±1.27^2^	4.06±1.38^1^	3.75±1.33^3^
Religious acceptance	4.65±1.43^2^	5.03±1.42^1^	3.16±1.70^3^
Encouragement	4.70±1.26^2^	4.93±1.36^1^	3.74±1.50^3^

1,2,3 -ranking; mean scores, low: 1–2.99, moderate: 3.0–5.0, high: 5.01–7.0.

GM salmon was perceived as being moderately beneficial (mean score of 3.90), moderately risky (mean score of 4.07), and moderately acceptable from the respondents' religious perspectives (mean score of 4.65), and was moderately encouraged (mean score of 4.70) by the Malaysian respondents (Table 5). The level of support for GM salmon among the Malaysian stakeholders was slightly more positive than that of other stakeholders worldwide. Grimsrud et al. [32] reported that consumers in Norway needed a 56% discount to willingly accept GM salmon over non-GM salmon. A more worldwide study carried out by Menozzi et al. [14] indicated that the experts and producers in their study believed that the acceptance of GM salmon by consumers worldwide was below the mid-point score of 5.0 (mean score of 2.43) while the mean score for the acceptance by producers worldwide was only 2.29.

Comparing GM salmon that involves same-species gene transfer in fish (*Chinook* salmon's growth gene into *Atlantic* salmon) with gene transfers in plants, it was found that GM salmon was less encouraged than plant-to-plant gene transfer in Malaysia [44] but was more acceptable than the transfer of a higher animal's gene into a plant [42] (Table 5). This pattern of acceptance can be explained by the balancing relationship of the factors determining the encouragement of a GM product (Figure 1). If the application is perceived as more acceptable from the respondents religious perspective, the risk is rated as lower and the benefits as higher, and the GM product is considered more familiar, which in the end translates to the product being more encouraged by the stakeholders.

### Determining factors

Figure 1 shows the final structural model of public attitude towards GM salmon. The model have a good fit with χ^2^/df = 1.565, a CFI of 0.954, a GFI of 0.902, a TLI of 0.948 and an RMSEA of 0.037 with a 90% confidence level in the range of 0.032–0.041. However, because a model generation strategy was used in this study, it must be acknowledged that the resulting model is in part data driven, but efforts have been taken to include only substantively meaningful modifications that do not contradict existing theory as recommended by Hair et al. [108], [110]. Various fit indexes have been chosen to adhere to standard practices in SEM and to account for sensitivity of SEM to the sample size and model misspecification. The chi-square test was carried out as it is considered essential in SEM studies [120] but, as expected, there was no significance owing to the large sample size. Bentler and Bonnet [121] and Joreskog [122] highlighted this issue where the chi-square statistic always rejects the model when large samples are used. The GFI was also reported to measure the extent to which the specified model is able to reproduce the sample covariance matrix [123]. Although the GFI has been widely used in the SEM literature, it has been reported to be insufficiently and inconsistently sensitive to model misspecification and strongly affected by sample size [124]. To address the impact of sample size on the model, normed chi-square (χ^2^/df) and incremental fit indexes such as the CFI [125] and TLI [126] were included. As proposed by Hu and Bentler [127] and MacCallum and Austin [128], the RMSEA is also embraced in this study to address any model misspecification problem; additionally, it has been proved to be able to generate an appropriate conclusion on the model quality and the availability of confidence interval that can provide important information about the precision of the estimate of fit [127]. Despite the effort taken to address SEM sensitivity, it is only appropriate to acknowledge the limitation of this model with respect to sampling effects, measures and occasions. The model in Figure 1 is valid in reflecting the Malaysian stakeholder attitude towards GM salmon, and the generalization of this model beyond this population is uncertain as the respective populations for the stakeholders involved were mostly unknown. The model was generated using a combined sample of various stakeholder representatives owing to the limitation of getting more respondents to represent categories of stakeholders such as producers, policy makers, NGOs and religious experts and because the respective populations of the stakeholders involved were mostly unknown. Nonetheless, the model is able to provide an initial picture of the important factors influencing the Malaysian stakeholder attitude towards GM salmon. In future work, this model should be cross-validated with more specific stakeholder groups. With respect to measures, the model is valid using the measured variables chosen in this study. Several steps have been taken into consideration with regard to the choice of indicators to represent latent variables. The indicators were developed according to the results of past studies and, when available, existing indicators were used. Standard testing for the validity and reliability of the measures were carried out and only good indicators were retained. The effect of occasions of measurement should be noted. The effects of the identified variables may vary over time and further research should be carried out for different time lags to better understand the influence of time.

The model in Figure 1 shows that perceived risk and religious acceptance are found to be the two most important direct predictors for the encouragement of GM salmon. Perceived risk is negatively associated with religious acceptance (β = −0.37) and encouragement (β = −0.28), while religious acceptance is positively associated with encouragement (β = 0.27). This result indicates that the Malaysians public's support towards GM salmon is mainly determined by their risk perception and religious acceptability. Only when GM salmon is considered as having low risks, it will be more acceptable from their religious point of view and translates to it being more encouraged. This finding is in contrast with the Malaysians attitude towards GM crop [21] and the Taiwanese attitude towards GM foods [49]. For GM crop, perceived benefit is the strongest predictor of its encouragement. It seems that the Malaysians are more concerns about the genetic modification of animal compared to plant. The experts in Menozzi et al. [14] study believed that GM salmon has more risks than benefits. Salmon farming have been identified by as the cause for diseases, pollution from waste effluents and pressure on wild fish stocks [129], [130]. For issue that they are more worried, they use perceived risk and moral consideration (religious acceptance) as determinants of their support. When they perceived GM salmon as risky, they are also not accepting of GM salmon from their religious perspectives. Malaysians identify themselves as being highly religious, where religion plays an important role in their day-to-day decision making processes [35]. Amin et al. [21] have also highlighted the importance of religiosity in their assessment of the general promises and concerns of modern biotechnology. Majority of the Malaysian respondents are Muslims whose views are governed by the Islamic law (*syariah*) [131]. For a GM food to be accepted by the Muslims, the food must be proven safe to the five purposes of *syariah*: preservation of religion, health, progeny, intelligence and wealth. The religious acceptance variable introduced in the model is a new factor that has not been included in other attitude towards GM food models. Therefore in countries like Malaysia and those with a similar culture, it is important to consider religious perspectives on GM food.

Although risk perception emerged as the dominant predictor of support for GM salmon in Malaysia, benefit still play a significant role in the Malaysians' attitude. As can be seen in Figure 1, perceived benefit is significantly associated with the encouragement of GM salmon (β = 0.14). The presence of clear benefits of GM salmon to the Malaysian public will enhance their support. This is in line with Brehdahl's research [132] which concluded that consumers attitude to GM foods is determined by the weighing up of their perceived risks and benefits. Both are determinants of attitude towards GM foods but their weightage differ according to different GM products. Surprisingly in this study, there was no significant relationship between perceived risk and perceived benefit of GM salmon. Although many studies reported an inverse relationship between the two variables and suggested that risk and benefit perceptions might represent the same feature [20], [24], [133] but Traill et al [134] found that risk and benefit perceptions are not perfectly correlated and suggested separate measurement. The finding of this study suggests that in a culture where the role of religion plays an important part in decision making, the stakeholders assessed perceived risks and benefits independently. This confirms the suggestion by Bredahl [19] and Connor and Siegrist [23] that there are cross-cultural differences in consumers' perception of the risks and benefits of GM foods.

Familiarity is positively associated with religious acceptance (β = 0.52) and encouragement of GM salmon (β = 0.16). When GM salmon is perceived as more familiar by the Malaysian respondents, it would be more acceptable from their religious perspective and is more encouraged by them. This finding is also supported by previous research on the public perception of GM technology. Italian consumers who claimed to be more familiar with GM food were also found to have positive attitudes towards the food [72]. Bredahl [135] also reported that perceived knowledge of genetic modification played a significant role in the acceptance of GM foods. Knowledge has been found to be an important factor in positive attitudes towards food among US, UK and French consumers [69]. However, it should be noted that the variable familiarity in this study consisted of two items reflecting personal control: whether it is easy to identify GM goods and whether it is easy to judge whether it is good or bad to consume GM food. In most studies related to attitude towards GM foods, objective knowledge have been tested [20], [49], [65]. This study affirmed the recommendation by House et al. [69] and Costa-font et al. [20] that subjective knowledge is the more important determinant for public acceptance of GM foods. However in addition to being subjective, this study has proved that it is not their perception of the “content knowledge” that is important but rather their perception of being able to differentiate whether the food is good or bad and whether they have control whether to eat them or not. The variable familiarity or ‘control’ has been frequently included in risk perception of different kind of hazards related to technology, life styles, environmental issues [56], [58], [70], [71] but not very frequently associated with GM foods. Genetic engineering is relatively a new technology that many people are not familiar with. Therefore there is a need to enhance the Malaysian people's familiarity with GM foods that can be achieved through knowledge enhancement in order for them to make informed judgement about GM foods. However in the case of GM food, the process of acquiring knowledge is not straightforward [20]. Costa-Font et al. [20] recommended three interrelated elements that must be considered: substantial reliable and accurate content, trust in information sources and the communication of the information. Scientists, being perceived as trustworthy by the public can play a prominent role in supplying accurate and reliable information to the media in Malaysia. The provision of labeling can provide accurate information and increase individual perception of personal control over the consumption of GM food [136]. From the model in Figure 1, it can be seen that the need for labeling is positively related to familiarity (β = −0.15). The respondents who stressed the high need for labeling of modern biotechnology products were those who were less familiar with GM salmon. Currently labeling of GM foods in Malaysia is not mandatory. Nevertheless it is encouraged that the GM foods industries to label their products to be marketed in Malaysia in order to increase public confidence in the product safety. When food industries provide consumers with the feeling of control over their food choices, confusion about what and how to choose will diminish and consumers will be more comfortable and confident in accepting GM food.

The Malaysian public general attitude and values were found to have influences on their attitude towards GM salmon. Among the general attitudinal variables, general promise of biotechnology was the only variable that had direct positive association with encouragement of GM salmon (β = 0.28). It was also positively associated with religious acceptance (β = 0.26) but negatively associated with perceived risk (β = −0.21; Figure 1). When the Malaysian respondents perceived the general promises of modern biotechnology as high, they also tended to regard GM salmon as less risky and were more accepting of GM salmon from their religious perspective which translated to them being were more encouraging of the product. In order for products of GM technology to be accepted, people must have a positive image of the technology in general. This association between process and products of GM technology has been explained by Bredahl [19]. On the other hand, general concern was positively associated with perceived risk (β = 0.29). Those who perceived modern biotechnology as having more concerns tended to perceive products of biotechnology such as GM salmon as highly risky too. The results of this study suggest that the Malaysians' broad general perception about biotechnology have direct effect on how they perceived GM salmon. It is expected that not all citizens of Malaysia would have a good understanding of specific applications of modern biotechnology but the informed public would have some general perceptions of biotechnology due to the cumulative effect of media coverage [45].

Individual values have been reported to affect their attitude towards a product [20], [64]. In this study, the factor of threatening the natural order of things was positively associated with general concern (β = 0.52) and perceived risk (β = 0.25; Figure 1). This result reveals that when the Malaysian respondents viewed modern biotechnology as highly threatening the natural order of things, they tended to regard it as having high concerns and its products, such as GM salmon, were also perceived as highly risky. GM technology has often been associated with interference with nature [51] and being seen as playing God [74]. Malaysians claimed that they are highly religious [35]. It is expected that people who are highly religious would be more concerns on the moral aspects of GM technology. Sjoberg [51] noticed a strong positive correlation between interfering with nature and perceived risk of genetic engineering. This study proves that moral aspects of modern biotechnology had significant influence on how people perceive specific application and product of GM technology such as GM salmon. The influence of values on attitude formation is supported by the ‘means-end’ theory which links product perceptions to consumers' values [20].

Confidence on regulation was positively associated with familiarity (β = 0.32) and perceived benefit (β = 0.28). The Malaysian respondents who were more confident in the regulation of GMOs tended to be more familiar with GM salmon and perceived it as having high benefits. This result is supported by earlier researchers. Gutteling et al. [137] reported that the Dutch public who have higher trust in the governance of GM food tended to be more positive towards GM foods. Trust in key players, such as scientists and the government have also been shown to be related to acceptance of GM technology [24], [138], [139] and its benefits [20], [140]. Confidence in regulation was also positively correlated with patenting (Figure 1). The Malaysian respondents who had high confidence in the regulation of modern biotechnology also supported the patenting of modern biotechnology products by the industries. Because of the positive influence of confidence on regulation on benefit perception, it is important that the Malaysian government be transparent and highly responsible in regulating the development and safe use of GM foods in Malaysia. Improper use of GM technology can be avoided with the presence of adequate regulations and effective monitoring systems. Once proper safety assurance mechanism is in place, it will build up public trust on the government and its governance and will increase their confidence to accept GM foods.

Societal value was positively associated with religious acceptance (β = 0.11) but negatively associated with perceived risk (β = −0.12). The Malaysian public who were more inclined towards materials tended to perceive GM salmon as less risky. The association between materialistic value and perceived risk is not unique to the Malaysian society as earlier researchers in western countries have proven that societal value exerted considerable influence on both perceived risk magnitude and acceptance of technological risks [141]. Gaskell et al. [24] reported that supporters of GM foods in Europe were those who believed in free market economic values, while those who were more concerned about the nature and environment were the rejecters. However it is interesting to note the association between materialistic value and religious acceptance, confidence on regulation and the need for labeling which has not been studied before. From Figure 1 it can be deducted that the Malaysians who were more materialistic will be more accepting of GM salmon from their religious point of view. This category of people will have higher confidence on gene technology regulation and stress less need for labeling of GM products.

Attitude to patenting was positively associated with general promise (β = 0.36). The Malaysian respondents who were highly supportive of the patenting of modern biotechnology products also perceived modern biotechnology as having high general promise. This factor has not been included in earlier studies. Even though there has been issues of patenting of gene technology [53], this study shows that the Malaysian people are more open minded in nature. They acknowledged the rights of the scientists and industries to patent their innovation and to cover the developmental costs of GM products. On the other hand, patenting was also positively correlated with the need for labeling. Although the Malaysian people recognized the patenting rights of the GM food developers, they insisted on their rights to information on the food that they eat.

The need for labeling was negatively associated with familiarity (β = −0.15). The Malaysian respondents who stressed a great need for labeling of modern biotechnology products were those who were less familiar with GM salmon. The influence of this factor on attitude to GM foods has not been tested in past studies. The need for labeling was also found to be positively associated with general promise (β = 0.22) and general concern of modern biotechnology (β = 0.31). This finding seems to reflect the main function of labeling in providing information either related to the benefits of the ingredients labeled or vice versa. Wansink and Kim [83] stressed the role of labeling as a signal of product safety. Some consumers might use labeling to avoid GM foods, but others may interpret labeling as the manufacturer's evidence for a product's safety. Once people felt confidence on the safety of GM foods, they will perceive the technology as promising, have less concerns and will be more accepting of them. Past study has shown two different effect of labeling. Some studies showed no change in consumers' attitude by increased product information [136], [142], [143]. On the other hand, other studies quoted a positive effect of giving additional information that involve clear consumer benefits [46], [144].

## Conclusion

In Malaysia, GM salmon was found to be moderately encouraged by stakeholders. The acceptance of GM salmon is determined by the balancing of the factors identified from the structural equation model developed in this study. The most significant finding in this study is the identification of specific application-linked perceptions about the religious acceptability as an important determinant of the encouragement of GM salmon besides perceived risks. It is important to highlight that for a country like Malaysia where religion plays an important part in their daily life and decision making, the factor religious acceptance should be seriously considered and assessed. The failure to do this will results in incomplete consideration of their major concerns related to GM foods. It is suggested that more in-depth studies on religious perspective of GM foods should be carried out to understand the reasoning behind the acceptance or rejection of GM foods.

There is a need to consider the balancing role of risk and benefits. In a country where religion plays a major role in decision making and when a GM application raises more concerns, perceived risk is a more prominent factor that needs to be look into in contrast with a GM application that have less concerns such as GM plant, perceived benefits will be the more dominant factor. The results of the study also suggest subjective knowledge (familiarity) that relates to personal control is more important than objective knowledge in relation to the acceptance for a complex issue such as GM foods. The factor of familiarity, which has rarely been included in attitudinal studies on GM foods, should be considered for inclusion in future studies.

Encouragement of GM salmon involves interplay between general attitudinal and value factors such as the general promise of and concerns about biotechnology, confidence in regulation, and societal values, which confirms the findings of earlier research on GM foods. Additional general schemas such as attitude to labeling and patenting and threatening the natural order of things, which have rarely been included in past studies, have been shown to shape attitudes towards GM salmon. These factors should also be considered in future research to understand their role in shaping public acceptance of GM foods.
